# POST-POLIO SYNDROME – SOMATOSENSORY DYSFUNCTION AND ITS RELATION TO PAIN: A PILOT STUDY WITH QUANTITATIVE SENSORY TESTING

**DOI:** 10.2340/jrm.v56.26192

**Published:** 2024-06-25

**Authors:** Daniel DAHLGREN, Kristian BORG, Eva MELIN

**Affiliations:** 1 Department of Rehabilitation Medicine, Danderyd University Hospital, Stockholm, Sweden; 2 Department of Clinical Sciences, Karolinska Institutet, Stockholm, Sweden

**Keywords:** postpoliomyelitis syndrome, post-polio syndrome, pain, somatosensory disorders, quantitative sensory testing

## Abstract

**Objective:**

To explore and characterize somatosensory dysfunction in patients with post-polio syndrome and chronic pain, by conducting examinations with Quantitative Sensory Testing.

**Design:**

A cross-sectional, descriptive, pilot study conducted during 1 month.

**Subjects/patients:**

Six patients with previously established post-polio syndrome and related chronic pain.

**Methods:**

All subjects underwent a neurological examination including neuromuscular function, bedside sensory testing, a thorough pain anamnesis, and pain drawing. Screening for neuropathic pain was done with 2 questionnaires. A comprehensive Quantitative Sensory Testing battery was conducted with z-score transformation of obtained data, enabling comparison with published reference values and the creation of sensory profiles, as well as comparison between the study site (more polio affected extremity) and internal control site (less affected extremity) for each patient.

**Results:**

Derived sensory profiles showed signs of increased prevalence of sensory aberrations compared with reference values, especially Mechanical Pain Thresholds, with significant deviation from reference data in 5 out of 6 patients. No obvious differences in sensory functions were seen between study sites and internal control sites.

**Conclusion:**

Post-polio syndrome may be correlated with a mechanical hyperalgesia/allodynia and might be correlated to a somatosensory dysfunction. With lack of evident side-to-side differences, the possibility of a generalized dysfunction in the somatosensory system might be considered.

LAY ABSTRACT

A significant number of polio survivors develop post-polio syndrome later in life, a condition characterized by increased weakness, fatigue, and pain. As the polio virus affects only specific parts of the nervous system, typically the sensory signalling pathways do not become affected. However, there are some reports suggesting that these patients may more often suffer from disturbed sensory functions compared with otherwise healthy subjects of the same age. The objective of this study was to explore this phenomenon in patients with post-polio syndrome and chronic pain with Quantitative Sensory Testing, a method more precise than the normal bedside examination. The study showed that post-polio patients in fact appear to have an overrepresentation of sensory abnormalities, and one may speculate that this might be a contributing factor in the development of chronic pain.

Poliomyelitis can result in various degrees of paresis through affection of lower motor neurons in the anterior horn of the spinal cord (1). In most parts of the world acute polio has been eradicated but new cases still occur in defined areas (2).

The initial paresis may be transient or in various degrees persistent after the acute phase. After years of stable disease, about 20–85% develop post-polio syndrome (PPS) (1, 3). PPS typically entails progressive muscular weakness and atrophy, cold intolerance, mental and physical fatigue, and pain (1, 3, 4). The cause of PPS is not completely understood but there is evidence suggesting ageing, inflammatory processes, muscle overuse, and concomitant disorders as contributing (1, 3–5).

Even though poliomyelitis affects lower motor neurons, somatosensory symptoms have been described (6). In a Danish cohort, self-reported pain and sensory symptoms were significantly more common in PPS patients (76.0% and 55.2% respectively), compared with controls (38.4% and 20.4% respectively), and survivors of poliomyelitis without PPS (36.7% and 17.2% respectively) (7). There are indications of impaired somatosensory function, such as mechanical and heat-induced allodynia, pathological somatosensory evoked potentials (SEP), and increased prevalence of restless legs syndrome (8–12). Human post-mortem studies and animal studies have shown inflammatory and degenerative changes in parts of the central nervous system as well, which are potentially responsible for these dysfunctions (6, 8, 13). Other mechanisms may be disturbances in spinal interneuronal structures as well as in ascending sensory pathways, or secondary impairments due to muscular atrophy and orthopaedic deformities, such as peripheral nerve injury and nerve entrapment (8, 14).

The pain associated with PPS is often multifocal and widespread (1, 4, 15, 16). Patients typically report a musculoskeletal ache worsened by physical activity and eased by rest (15). The pain has been described as nociceptive (17). However, according to the IASP (International Association for the Study of Pain) 2011 definition of pain, nociceptive pain requires a “normally functioning somatosensory nervous system” (18). This causes the question of whether pain in PPS could also have neuropathic components, after all.

Quantitative Sensory Testing (QST) is a semi-quantitative, non-invasive, standardized method for evaluating the somatosensory profile of patients with regard to tactile, thermal, vibratory, and pain-associated sensory functions (19). It is used as a complement to the clinical examination, but as there are standardized QST protocols the results allow for follow-up and comparisons against published normative values with regard to age, gender, and test site (20–23). QST is a well-established method in the diagnosis of sensory neuropathies and neuropathic pain (19, 24). It has been used in exploratory studies of a variety of conditions such as osteoarthritis, chronic back pain, and fibromyalgia (25–27). In one study, Kumru et al. (9) detected lowered mechanical pain thresholds and heat-induced pain thresholds in polio-affected limbs, but no differentiation between patients with or without PPS was made.

The aim of this study was to examine patients with PPS and associated pain with QST to investigate signs of a typical sensory profile for PPS, and a relationship between QST-derived sensory abnormalities and motor dysfunction, as well as the distribution and characteristics of pain. If such correlations exist, it could lead to better understanding of the mechanisms behind the pain associated with PPS.

## PATIENTS AND METHODS

### Study design

The current study was designed as a cross-sectional, descriptive, pilot study.

### Patient selection

Patients were non-randomly included from a previously established cohort of 16 patients participating in a study of Pain and Fatigue in PPS at the Postpolio Outpatient Clinic at Danderyd Hospital (28). All 16 patients’ medical records were searched with regard to inclusion and exclusion criteria. Inclusion criteria were known chronic pain and diagnosed post-polio syndrome according to the March of Dimes criteria (see Table I) (29). Exclusion criteria were other conditions that might affect the result of QST, or not being eligible to participate for other reasons according to the study investigators. Six patients met these criteria and all were contacted and included after consent was given. Demographic data are presented in Table II. 10 patients were excluded due to lumbar spinal stenosis (*n* = 1), lumbar disc hernia (*n* = 1), under investigation for polyneuropathy (*n* = 2), confirmed polyneuropathy (*n* = 2), restless legs syndrome (*n* = 1), or not being able to participate due to psychological or cognitive factors (*n* = 3).

**Table I:** Post-polio syndrome criteria according to the March of Dimes (29)

1	Prior paralytic poliomyelitis with evidence of motor neuron loss, as confirmed by history of the acute paralytic illness, signs of residual weakness and atrophy of muscles on neurological examination, and signs of denervation on electromyography
2	A period of partial or complete functional recovery after acute poliomyelitis, followed by an interval (usually 15 years or more) of stable neurological function
3	Gradual or sudden onset of progressive and persistent new muscle weakness or abnormal fatigability (decreased endurance), with or without generalised fatigue, muscle atrophy, or muscle and joint pain. (Sudden onset may follow a period of inactivity, or trauma or surgery.) Less commonly, symptoms attributed to postpolio syndrome include new problems with breathing or swallowing
4	Symptoms persist for at least a year
5	Exclusion of other neurological, medical, and orthopaedic problems as causes of symptoms

**Table II:** Demographic data

	Patient no.						Mean
	1	2	3	4	5	6	
Age (years)	66	71	68	73	65	70	69
Gender (male/female)	M	F	F	F	M	F	–
Years since acute poliomyelitis	64	66	66	69	63	69	66
Years since PPS diagnosis	23	30	30	24	25	23	26

Previous neurophysiological examinations such as electroneurography (ENeG), electromyography (EMG), and, if performed, Macro-EMG were reviewed in order to confirm the presence of late polio neuromuscular changes in included patients (30).

All examinations were conducted at the Pain Outpatient Department at Karolinska University Hospital Solna, Stockholm, Sweden during a period of 1 month. The clinical examinations and pain anamnesis were all performed by the same medical doctor (n. D.D.) QST was performed by a specialized nurse certified in the procedure.

### Pain anamnesis and questionnaires

Anamnestic information regarding current and previous pain was obtained, as well as pain distribution and ratings according to the Numeric Rating Scale (NRS) 0–10, 0 meaning *no pain* and 10 meaning *worst possible pain*. A pain drawing was performed.

Two different questionnaires aiming to screen for neuropathic pain were employed: Doleur Neuropathique 4 questions (DN4) and painDETECT questionnaire (PD-Q). Both these questionnaires are validated and show a high grade of sensitivity and specificity in screening for neuropathic pain (31). For this study we used the Swedish versions (32). DN4 consists of 7 questions addressing symptoms associated with neuropathic pain as well as 3 questions answered by the investigator after the bedside sensory examination. A score of ≥ 4 points make neuropathic pain likely and < 4 points unlikely (33). PD-Q consists of 7 questions addressing symptoms associated with neuropathic pain, a pain drawing, and a question regarding the temporal profile of the course of the pain, as well as self-rating of pain. A score of 0–12 points suggests that a neuropathic pain component is unlikely, 13–18 points possible, and 19–38 makes a diagnosis of neuropathic pain probable (34).

### Clinical bedside examination

The patients underwent a clinical examination with a manual motor test of muscles in the upper and lower extremity bilaterally, a test of tendon reflexes, and inspection of muscular atrophy. Bedside examination of sensory functions was conducted on all extremities; for light touch a standardized brush was used (Brush-05, SENSELab™, Somedic Sales AB, Hörby, Sweden), for pinprick a needle, for temperature sensation a hot and cold metallic roller was used (SENSELab™, Rolltemp, Somedic Sales AB, Hörby, Sweden), and for vibration using a vibration fork.

### Quantitative Sensory Testing

Selected QST parameters (presented below) were set according to the protocol published by the German Research Network on Neuropathic Pain (DFNS) and with the method of limits (19, 22, 23). All patients were examined on all 4 extremities for all presented modalities at the thenar region of the hand on the upper extremity and on the anterior part of the lower leg (L5 dermatome) or lateral part of the foot (S1 dermatome) on the lower extremity.

The Mechanical Detection Threshold (MDT) was assessed using a set of modified von Frey-hair exerting forces between 0.25 and 512 mN (Optihair von Frey Filaments, Marstock nervtest, Dr. Fruhstorfer, Marburg, Germany). The Mechanical Pain Threshold (MPT) was determined using a set of 7 weighted, calibrated, pinprick stimulators exerting forces between 8 and 512 mN. The Cold Detection Threshold (CDT), Warm Detection Threshold (WDT), Cold Pain Threshold (CPT), and Heat Pain Threshold (HPT) were measured using a contact thermode with an area of 12.5 cm^2^ (MSA Thermotest™, Somedic Sales AB, Hörby, Sweden) starting from skin temperature. The Vibration Detection Threshold (VDT) was measured using a Rydel–Seiffer tuning fork (64 Hz, 8/8 scale). The Pain Perception Threshold (PPT) was measured with a pressure gauge device with a 1 cm^2^ pressure surface (Force Dial^TM^ FDK 20, Wagner Instruments, Greenwich, CT, USA). The results from the PPT test were converted from kg/1 cm^2^ to kPa (by a factor of 98,0665) to enable comparison with published normative values.

### Data analysis

From the neurological examination (muscle strength, tendon reflexes, and presence of atrophy) the study investigators identified for each patient 1 (*n* = 5) or 2 (*n* = 1; Patient no. 2) extremities with a more pronounced neuromuscular affection (called *study sites*) compared with their contralateral sides (called *control sites*) when QST data were analysed. In a similar manner, we identified for each patient the distribution and intensity of pain for each extremity.

All statistical analysis was undertaken using Microsoft Excel (Microsoft Office 365; Microsoft Corp, Redmond, WA, USA) and all data calculations presented were made according to the DFNS protocol (22, 23). From the raw data arithmetic and/or geometrical mean values were calculated. CDT, WDT, MDT, MPT, and PPT mean values were logarithmically transformed to achieve a normal distribution. For the data achieved from MDT and MPT, which results in 5 test-pair results, the first 2 test pairs were excluded as these could differ significantly from the following results.

The z-scores were calculated ((Mean_x_ – Mean_reference_)/SD_reference_), which enables the mean values obtained to be weighted against published reference mean values and standard deviations (21). This also enables comparisons between patients, even though they differ with regard to sex, age, and test site. The algebraic sign of the z-scores was changed for CDT, WDT, HPT, MDT, MPT, and PPT to make a positive z-score indicate *gain of function* (i.e., hypersensibility) and a negative z-value indicate *loss of function* (i.e., hyposensibility). A z-score < –1.96 and > 1.96 was considered a significant deviation (95% confidence interval). With these z-scores we were able to produce sensory profiles for every patient (23).

### Ethical approval

Ethical approval was obtained by the regional Ethical Review Board in Stockholm, Sweden Dnr 2020-01794.

## RESULTS

### Clinical bedside examination

All test sites enrolled as study sites (*n* = 7, including two test sites for Patient no. 2) presented signs of paresis, hypo-/areflexia, and the presence of muscular atrophy. Test sites enrolled as control sites in all cases also showed these signs but to a lesser extent.

Two out of 5 patients had a history of subjectively impaired sensory function with no history or clinical features suggestive of other neurological condition (Patient no. 1 was not counted due to signs of a previous unknown diabetes-associated painful neuropathy in the 3rd to 5th digit on the right foot). Four out of 6 had a pathological bedside sensory examination with findings of hypesthesia for hot and cold temperature as the most common disturbance (4 out of 4) and in most of these cases (3 out of 4) symmetrically distributed in the lower extremities with a distal-proximal gradient. One patient (Patient no. 4) had a unilateral hypesthesia for all modalities at bedside examination in the right leg (study site) but normal results in the left leg.

### Pain

All patients reported significant pain during the last 4 weeks with a mean NRS of 6.7/10 at worst, but with signs of great variability over time (see Table III). All reported pain worsened on increased physical activity and was alleviated with rest. All reported intolerance to cold temperature with increased pain.

**Table III:** Pain ratings and questionnaires

		Patient no.						Mean
		1	2	3	4	5	6	
Pain questionnaires	DN4 (0–10)	6^a^	2	3	5	1	1	*3*
	PD-Q (0–38)	13^b^	12	10	9	8	5	*9,5*
Pain ratings, NRS (0–10)	Current pain	2	1	3	3	0	0	*1,5*
	Pain, strongest last 4 weeks	7	6	6	7	6	8	*6,7*
	Pain, mildest last 4 weeks	1	1	0	2	0	0	*0,7*
	Mean pain last 4 weeks	3	3	2	4	1	3	*2,7*

a Neuropathic pain likely.

b Neuropathic pain component possible.

NRS: Numeric Rating Scale; DN4: Doleur Neuropathique 4 Questions; PD-Q: painDETECT Questionnaire.

**Fig. 1:**
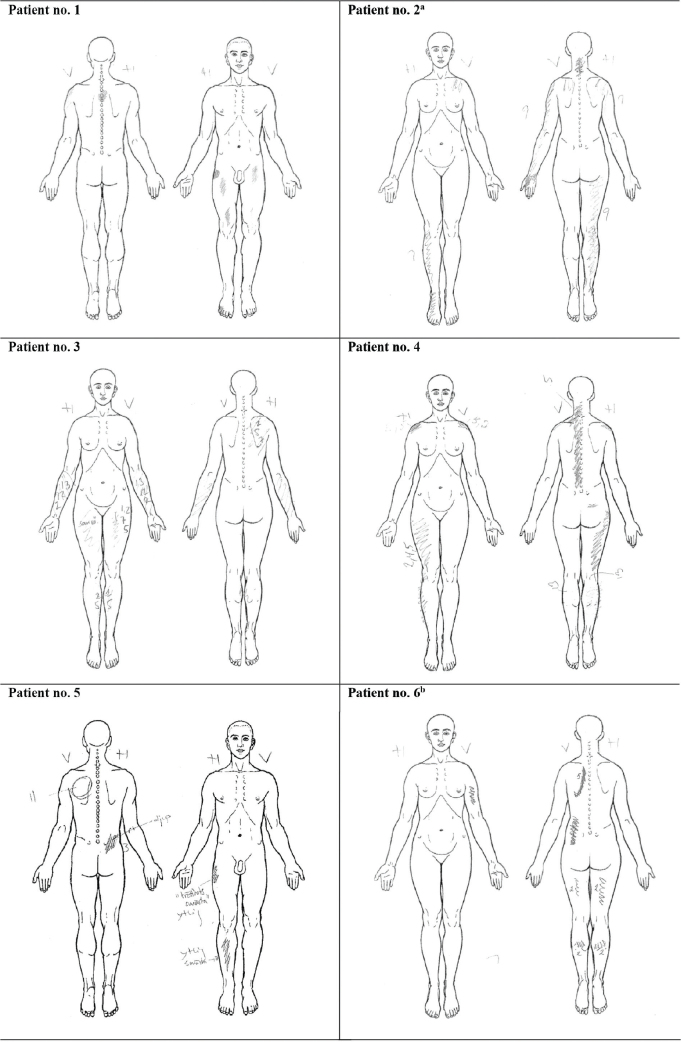
Pain drawings. ^a^ Left arm pain radiating from subacute, nociceptive shoulder pain and not related to long-lasting PPS pain (according to the patient). ^b^ Left arm pain marked in the drawing as it was more prominent at the time of examination, but reported by the patient that the pain is usually similar in both arms.

Pain drawings (presented in Fig. 1) were assessed, together with a thorough anamnesis. In some cases, the pain drawings were not completely concordant with pain anamnesis (see Fig. 1 footnotes). For most of the patients the pain was multifocal and to a great extent localized to body parts with neuromuscular affection. In 5 out of 6 patients the pain was worst or at least equally prominent in the extremity more affected by polio, but for Patient no. 5 the pain was more prominent on the contralateral side.

Screening for neuropathic pain with DN4 and PD-Q did not indicate the presence of neuropathic pain, except for Patient no. 1 whose results may indicate neuropathic pain (DN4) or a possible neuropathic pain component (PD-Q), but these results were derived from an isolated, burning pain in the right foot probably due to the previously mentioned diabetes-associated neuropathy, and not related to the PPS-associated pain.

### QST

QST z-scores were calculated for all of the study sites and their respective internal control sites. Mean values and z-score for each parameter are presented per patient in Table IV. All patients had at least equally prominent pain in the most neuromuscular affected extremity, except Patient no. 5 who reported more pain in the right leg but more prominent paresis and atrophy in the left leg. In this case we chose to report these as their respective internal controls, depending on whether one wishes to use pain or motor function as study site.

**Table IV:** Data from Quantitative Sensory Testing (QST)

	Site	Patient no.													
		1		2				3		4		5		6	
		Study site	Control site	Study site	Control site	Study site	Control site	Study site	Control site	Study site	Control site	Study site	Control site	Study site	Control site
		LE right	LE left	UE right	UE left	LE right	LE left	LE left	LE right	LE right	LE left	LE right	LE left	UE right	UE left
CDT (°C from baseline)	Mean (log)	1.076	0.176	0.146	0.204	0.740	0.322	0.415	0.591	0.230	0.204	0.146	2.162	–0.046	–0.097
	*Reference mean (SD)* ^a^	*0.616 (0.266)*		*0.187 (0.271)*		*0.377 (0.298)*		*0.377 (0.298)*		*0.377 (0.298)*		*0.616 (0.266)*		*0.187 (0.271)*	
	z-score^b^	–1.729	1.654	0.151	–0.063	–1.218	0.185	–0.128	–0.718	0.493	0.581	1.767	2.162	0.860	1.048
WDT (°C from baseline)	Mean (log)	1.158	1.210	0.279	0.279	1.072	1.196	0.839	0.681	0.740	0.839	0.934	1.228	0.204	0.079
	*Reference mean (SD)* ^a^	*0.803 (0.237)*		*0.368 (0.211)*		*0.657 (0.222)*		*0.657 (0.222)*		*0.657 (0.222)*		*0.803 (0.237)*		*0.368 (0.211)*	
	z-score^b^	–1.498	–1.717	0.422	0.422	–1.869	–2.430	–0.820	–0.108	–0.374	–0.820	–0.553	–1.793	0.777	1.370
CPT (°C)	Mean	10,000	10,000	11,467	13,500	10,000	12,700	10,000	11,700	10,000	10,000	25,800	16,500	13,933	12,433
	*Reference mean (SD)* ^a^	*11,19 (11)*		*8,58 (8,09)*		*9,12 (8,42)*		*9,12 (8,42)*		*9,12 (8,42)*		*11,19 (11)*		*8,58 (8,09)*	
	z-score	–0.108	–0.108	0.357	0.608	0.105	0.425	0.105	0,306	0,105	0,105	1,328	0,483	0,662	0,476
HPT (°C)	Mean	44.367	48.567	44.667	46.467	47.733	48.100	44.533	43,067	45,333	42,433	40,300	46,567	44,100	45,133
	*Reference mean (SD)* ^a^	*47.74 (1.55)*		*45.3 (2.24)*		*45.99 (1.99)*		*45.99 (1.99)*		*45.99 (1.99)*		*47.74 (1.55)*		*45.3 (2.24)*	
	z-score^b^	2.176	–0.534	0.283	–0.521	–0.876	–1.060	0.732	1,469	0,330	1,787	4,800	0,757	0,536	0,075
MDT (mN)	Mean (log)	1.355	1.003	–0.753	–0.702	0.602	0.753	0.201	0,602	1,907	1,455	1,104	1,054	–0,552	–0,652
	*Reference mean (SD)* ^a^	*0.263 (0.474)*		*0.241 (0.430)*		*0.388 (0.555)*		*0.388 (0.555)*		*0.388 (0.555)*		*0.263 (0.474)*		*0.241 (0.430)*	
	z-score^b^	–2.303	–1.561	2.312	2.193	–0.386	–0.658	0.337	–0,386	–2,737	–1,923	–1,774	–1,669	1,844	2,077
MPT (mN)	Mean (log)	0.753	1.154	0.853	0.753	0.753	0.753	1.204	1,505	2,860	2,860	0,853	1,154	0,753	0,753
	*Reference mean (SD)* ^a^	*1.967 (0.275)*		*1.769 (0.334)*		*1.673 (0.378)*		*1.673 (0.378)*		*1.673 (0.378)*		*1.967 (0.275)*		*1.769 (0.334)*	
	z-score^b^	4,415	2,956	2,743	3,042	2,434	2,434	1,241	0,447	–3,140	–3,140	4,051	2,956	3,042	3,042
VDT (x/8)	Mean	3,000	2,000	7,667	7,333	6,667	7,333	6,000	7,000	5,000	5,000	6,333	6,333	7,333	7,000
	*Reference mean (SD)* ^a^	*6.54 (1)*		*7.6 (0.51)*		*6.49 (1.51)*		*6.49 (1.51)*		*6.49 (1.51)*		*6.54 (1)*		*7.6 (0.51)*	
	z-score	–3.540	–4.540	0.131	–0.524	0.117	0.558	–0.325	0.338	–0.987	–0.987	–0.207	–0.207	–0.524	–1.176
PPT (kPa)	Mean (log)	2.699	2.911	2.544	2.488	2.924	2.926	2.832	2.594	2.321	2.353	2.685	2.444	2.423	2.531
	*Reference mean (SD)* ^a^	*2.678 (0.151)*		*2.65 (0.076)*		*2.675 (0.132)*		*2.675 (0.132)*		*2.675 (0.132)*		*2.678 (0.151)*		*2.65 (0.076)*	
	z-score^b^	–0.139	–1.543	1.395	2.131	–1.869	–1.902	–1.189	0.614	2.682	2.439	–0.046	1.550	2.987	1.566

a Reference values obtained from Magerl et al. (21).

b Algebraic sign converted.

UE: upper extremity; LE: lower extremity; CDT: Cold Detection Threshold; WDT: Warm Detection Threshold; CPT: Cold Pain Threshold; HPT: Heat Pain Threshold; MDT: Mechanical Detection Threshold; MPT: Mechanical Pain Threshold; VDT: Vibration Detection Threshold; PPT: Pain Perception Threshold.

One patient had a completely normal QST result, while the rest (83.3%) had sensory abnormalities in at least 2 different parameters, which is 2–3 times more frequent than in healthy subjects (35).

**Fig. 2:**
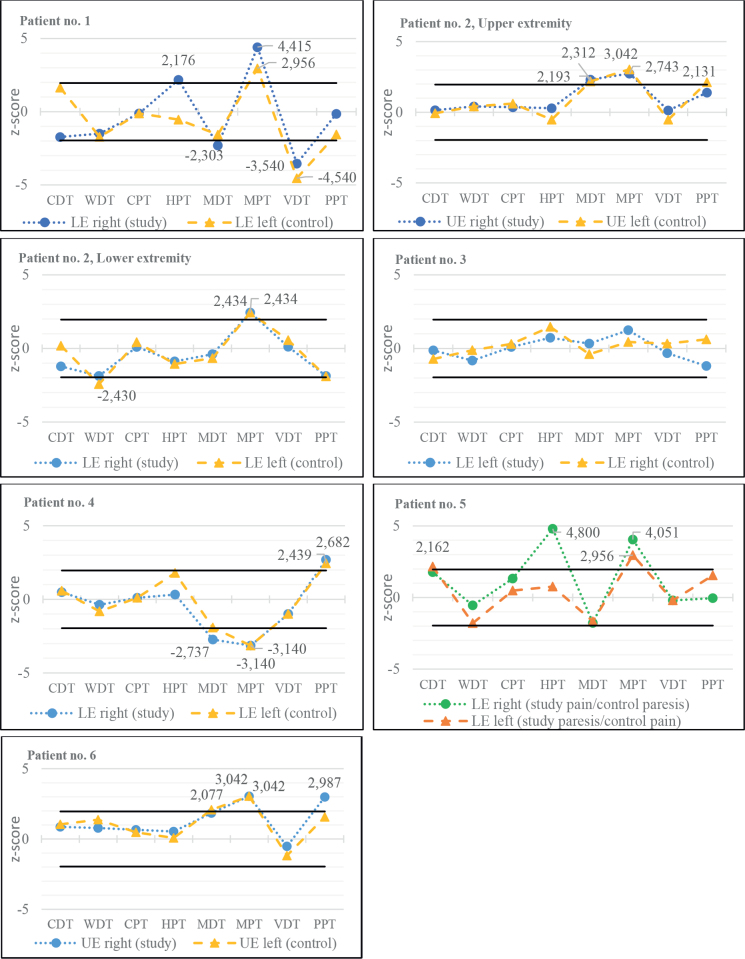
z-score sensory profiles of patients’ more affected limbs (study sites) and internal control sites (control). Solid, black lines indicating *+*1.96 SD and –1.96 SD respectively. UE: upper extremity; LE: lower rxtremity; CDT: Cold Detection Threshold; WDT: Warm Detection Threshold; CPT: Cold Pain Threshold; HPT: Heat Pain Threshold; MDT: Mechanical Detection Threshold; MPT: Mechanical Pain Threshold; VDT: Vibration Detection Threshold; PPT: Pain Perception Threshold.

The sensory profiles (see Fig. 2) revealed high consistency between the study sites and control sites for every single patient with a minority of parameters with a significant side-to-side difference, but great variability between the subjects’ individual profiles. The Mechanical Pain Threshold, MPT, showed a pathological z-score for 6 out of 7 profiles (5 out of 6 with gain of function, i.e., decreased pain thresholds) in both tested extremities, while the other parameters presented greater variability.

**Fig. 3:**
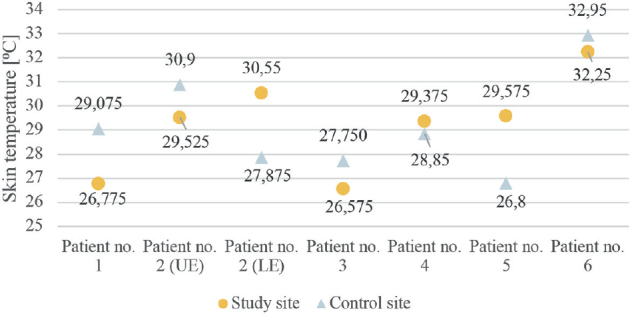
Skin temperature at study site and control site respectively. UE: upper extremity; LE: lower extremity.

As all patients reported intolerance to cold temperature and most of them (5 out of 6) reported sensations of being colder in their PPS-affected limbs, we compiled the skin temperatures obtained at the thermal QST tests without signs of an objectively stated difference between study and control sites (see Fig. 3).

## DISCUSSION

This descriptive pilot study presents sensory profiles derived from Quantitative Sensory Testing from 6 patients with post-polio syndrome and long-lasting pain. The sensory profiles showed great variability between the different patients with most z-score converted results within ±1.96 SD. However, 83.3% of the patients had at least 2 pathological QST parameters, which indicates that sensory abnormalities are more common than in healthy subjects. The most common altered parameter was the Mechanical Pain Threshold (MPT) with significant deviation from reference normative data in 6 out of 7 sites, in which *gain of function* (positive z-score) was the most common (5 out of 6). This might reflect the presence of mechanical allodynia/hyperalgesia mediated through Aδ- and C-fibres (36, 37). These findings appear to be consistent between the study sites and the internal control sites for most of the subjects, without obvious considerations of the degree of pain or paresis in their respective sites, while not being able to confirm the previous findings by Kumru et al. (9) with mechanical and thermal allodynia more prominent in the more polio-affected extremity. This might possibly indicate a more generalized overactivation of the pain signalling system, e.g., peripheral and/or central sensitization, seen in other chronic, non-malignant, pain conditions (18, 37), and corroborated by the finding of elevated expression of prostaglandins in skeletal muscle in patients with prior polio (5). However, even in strictly unilateral neuropathic pain conditions, such as postherpetic neuralgia, changes in the somatosensory function have also been described on the contralateral side (35). In all, the pathophysiological mechanisms behind the PPS-related pain and the possible somatosensory dysfunctions are still unclear, and to determine whether this reflects a more generalized affection of the central nervous system further studies are to be conducted.

A common and well-reported symptom in PPS is cold intolerance, also found in this study. One might have expected to find signs of, for example, a decreased Cold Pain Threshold (i.e., cold-induced allodynia), but the thermal QST tests (CDT, WDT, CPT, and HPT) were in almost all cases within the 95% confidence interval. Even the acquired skin baseline temperature did not verify a lower skin temperature on the more affected side, as we would have expected. However, this was not a predefined objective for this study, and therefore no considerations of test site and procedure were taken before the tests.

A better understanding of the underlying mechanisms behind PPS-associated pain is of great value in order to find an effective treatment strategy. In this study, screening with questionnaires (DN4 and PD-Q) did not indicate the presence of a neuropathic pain component consistent with previous findings. Kosek et al. (18) presented in 2016 the idea of nociplastic pain, i.e., pain conditions with signs of “altered nociceptive function” but without the phenomenology and structural conditions associated with neuropathic pain. If, for example, mechanical hyperalgesia/allodynia associated with PPS were to be confirmed in a future, larger study this might strengthen the theory of sensitization as part of the pain seen in PPS. This could possibly also give further insights into the fact that it appears to be more frequent in patients with PPS that they also fulfil the criteria of concomitant fibromyalgia with generalized musculoskeletal pain (4, 38).

Boshuis et al. (28) suggested the term “post-polio muscular pain” for the pain reported by PPS patients, a deep muscular pain not restricted to a specific dermatome or peripheral nerve. A sensory disturbance was found in the present study, which could indicate that the pain in PPS patients could be of neuropathic type. However, the findings from the questionnaires DN4 and PD-Q do not favour the presence of neuropathic pain. Thus, this further supports the presence of a “post-polio muscular pain”, a condition not quite fulfilling the criteria for either nociceptive or neuropathic pain.

The results of this descriptive pilot study need to be interpreted with caution for several reasons. The number of included patients is small and therefore we had no intention to achieve statistically significant results. We had limited possibilities to check for possible confounding concomitant disorders that may have influenced the test results. We only used previously published reference values and not data from a healthy control group. Neither did we compare patients with PPS with patients with prior poliomyelitis without a diagnosis of PPS. As 36–41% of healthy controls have at least one parameter outside the confidence interval in a full QST battery, we cannot with certainty state that the prevalence of sensory dysfunction in this study (83.3%) would not have been equally present in a healthy control group (35). The QST test site was standardized in all subjects but no consideration was made as to whether pain did occur at that site or not, which might also have influenced the results, especially with regard to the use of internal controls. Also, it would be of great interest to compare patients with PPS and non-neuropathic pain with patients with PPS and neuropathic pain with regard to QST, but such a study would need to include a significantly larger study cohort.

The patients were not randomly selected, and the study group was homogeneous regarding age and duration of PPS. This might reflect the fact that the patients in this cohort reported a mean current pain according to NRS as 1.5/10, which is lower than might be expected. One possible explanation could be the fact that self-reported pain tends to decrease with increasing age (7, 17, 39). A younger cohort, or a more heterogeneous group, might give other results. Another limitation is the fact that the patients did have neuromuscular affection (paresis, areflexia, and/or atrophy) in both the study site and internal control site, at least to some extent. To examine patients with strictly unilateral dysfunction could possibly lead us to better insights in the alterations in somatosensory functions seen in post-polio syndrome.

In conclusion, we present sensory profiles and pain characteristics derived from clinical examination and Quantitative Sensory Testing in patients with post-polio syndrome and associated chronic, multi-focal pain. The results may indicate the presence of an altered somatosensory function, especially mechanical hyperalgesia/allodynia. No obvious correlations were seen between degree of neuromuscular dysfunction, pain, and somatosensory aberrations, even though statistical correlation analysis was not conducted due to the limited number of subjects. The underlying mechanisms of these possible sensory alterations and their clinical relevance remains unclear, even if the results of the study point to the altered sensory function indicating that the pain found in PPS patients is not of a neuropathic type. It does not fulfil the criteria of nociceptive pain and one may speculate that the pain reported is a separate pain entity as previously suggested by Boshuis et al. (28). More studies involving QST could give further insights and future studies should include a larger cohort with a more heterogeneous population and a healthy control group to allow for statistical and correlation analysis in order to further determine the origin and relevance of sensory abnormalities associated with PPS.
